# Role of traditional healers in the management of microbial keratitis in eastern Nepal

**DOI:** 10.12688/wellcomeopenres.21241.2

**Published:** 2024-11-04

**Authors:** Sandip Das Sanyam, Reena Yadav, Abeer H. A. Mohamed Ahmed, Simon Arunga, Astrid Leck, David Macleod, Abhishek Roshan, Sanjay K. Singh, Sailesh K. Mishra, Jeremy J. Hoffman, Matthew J. Burton, Tara Mtuy

**Affiliations:** 1 Sagarmatha Choudhary Eye Hospital, Siraha, Lahan, 56502, Nepal; 2 International Centre for Eye Health, London School of Hygiene and Tropical Medicine, London, WC1E 7HT, UK; 3 Department of Ophthalmology, Mbarara University of Science and Technology, Mbarara, Western Region, 1410, Uganda; 4 MRC International Statistics & Epidemiology Group, London School of Hygiene and Tropical Medicine, London, WC1E 7HT, UK; 5 Nepal Netra Jyoti Sangh, Kathmandu, Nepal; 6 National Institute for Health Research Biomedical Research Centre for Ophthalmology at Moorfields Eye Hospital NHS Foundation Trust and UCL Institute of Ophthalmology, London, EC1V 9EL, UK

**Keywords:** microbial keratitis (MK), traditional healers, eye care, treatment, practices, traditional eye medicine (TEM)

## Abstract

**Background:**

Microbial Keratitis (MK) is a leading cause of corneal blindness due to infection and its consequences, with a higher incidence in resource-limited nations. Hospital-based patient records from different parts of Nepal suggest patients often use traditional eye medicine to treat MK. Traditional healers (TH) within the community are often the first point of care for MK management. Little is known of their practice, perceptions, and knowledge around MK management. We aimed to understand the role of traditional healers in the management of MK in south-eastern Nepal.

**Methods:**

A cross-sectional, mixed method, descriptive study was conducted in the Siraha district of Nepal. A total of 109 traditional healers consented to participate in a survey of knowledge, attitude, and practices. Some participants were also invited to participate in in-depth interviews and focus group discussions. Interviews and focus groups were conducted and recorded in the Maithili language by a native speaking interviewer and transcribed into English. Descriptive analysis was performed for the survey. Data saturation was considered the endpoint for qualitative data collection, and a thematic was analysis applied.

**Results:**

Traditional healers believe that infection of the eye can be caused by trauma, conjunctivitis, or evil spirits. They were unclear about differentiating MK from other eye conditions. They provided various types of treatment. Some were confident that they could treat severe ulcers that had not responded to medical therapy, while others thought treating larger diameter ulcers would be difficult. Although there were mixed responses in referring patients with MK, the majority of TH were willing to refer.

**Conclusion:**

In a weak health system, traditional healers may help address barriers to healthcare access and reduce delays to definitive care, upon integration into the formal health system and referral pathway.

## Introduction

Microbial keratitis (MK) is an infection of the cornea that can lead to permanently reduced vision, blindness or even loss of the eye. Globally, the most recent estimate suggests that 5.5 million people are bilaterally blind or have moderate or severe vision impairment (MSVI) and a further 6.2 million are blind in one eye from corneal pathology ^1^. MK is the most common condition responsible for monocular corneal blindness in low-income and middle-income countries (LMICs) ^2^. In low-income settings, particularly in tropical regions, a high proportion of these infections are caused by filamentous fungi, most commonly following traumatic corneal abrasion with vegetative matter, and are frequently seen in people involved in agriculture, including subsistence farmers ^3^. MK frequently results in monocular blindness, due to dense corneal scar after the ulcer is healed or a severe disease leading to loss of an eye. These events are regularly reported from LMICs in Africa and Asia ^4– 6^. Nepal has the highest reported incidence of microbial keratitis globally ^7^.

MK requires prompt diagnosis and intensive antimicrobial treatment to limit vision loss, however, standard diagnostic and treatment practices may not be followed. Barriers to accessing modern (non-traditional) medicines in many low-income settings, such as Nepal, include poverty, transportation challenges, limited availability of services, and political economy ^8, 9^. Poor access to formal health systems and preferences for traditional medical treatments have led people to seek traditional ways to treat eye conditions ^10^. In Nepal patients often initially use home remedies and/or seek care from traditional healers (TH), locally referred to as ‘ *Dhami’,* or from pharmacy shops ^11, 12^.

Reports from hospital-based studies suggest that 0.7–36% of patients have visited traditional healers to obtain treatment before attending a hospital for MK in Nepal ^6, 12, 13^. In a recent randomised controlled treatment trial of fungal keratitis in Nepal we found many patients had visited a TH before presenting to the eye hospital ^14^. These patients believed corneal ulcers may have occurred due to religious curses or witchcraft, and they thought that corneal ulcers could be treated by traditional healing methods. Most of the patients who visited a TH before coming to the eye hospital had a poor visual outcome ^11, 12, 15^.

With improvement in services and resources at health care facilities, and through community health programs, improved access and uptake of health services and allopathic medicine at the community level is anticipated. To the best of our knowledge, no study to date has sought to understand traditional healer practices in the management of MK in Nepal. This paper aims to explore current knowledge and experiences of traditional healers in the management of MK and ways to engage with them to improve referral of patients with suspected MK.

## Methods

### Study setting and participants

We conducted this mixed-methods descriptive cross-sectional study based out of Sagarmatha Choudhary Eye Hospital (SCEH), Siraha District, Nepal between February, and November 2021. Siraha District is in the south-eastern part of the Terai region (lowlands). The majority of the population follow Hinduism, they are mostly of Terai origin caste and involved in subsistence farming agriculture. There are approximately 500 THs in Siraha district; these formed the sampling frame for the different methods we used: a knowledge, attitude, and practice (KAP) survey, in-depth interviews (IDIs), and focus group discussions (FGDs).

We randomly selected thirty of the 112 community health centres in Siraha district. These were selected in equal proportions for Urban, sub-urban and rural communities, with geographical stratification. We contacted the selected health centres to help us identify the THs working in the area served by each health centre. Lists of traditional healers in the selected areas were compiled with support from each municipality and Village Development Committee (VDC) through their contacts, health centre records, and female community health volunteers (FCHV). The study team used the lists to make contact in person with the THs and identify the THs who were actively involved in the treatment of MK. Some of them were traced, while others could not be found or had died. No additional follow up was done for those that were not available. Altogether 109 THs were visited, invited to participate in the KAP survey, agreed and were recruited. Purposive sampling was used to recruit for IDIs and FGDs, based on the study team’s interactions with participants during the KAP survey. Those that expressed interest and willingness to discuss their practice and experience were invited to participate in IDIs and FGDs. Some of the participants who participated in IDI, showed their interest to participate in FGD and they were included for the discussion.

### KAP survey

Ophthalmic assistants and eye health workers from the study team conducted the KAP survey in Maithili language. The KAP survey included 15 questions covering knowledge, attitudes, and practises of managing suspected MK cases. Questions were developed and validated by a focus group of experienced researchers working on MK. The survey was pretested with a small number of traditional healers, and the tool was refined for reliability. Interviewer-administered, the questions were read aloud, and the answers given by THs were recorded in the survey record sheet. Thirteen questions had predefined multiple choice response options; however, these options were not read to the participants. One of the questions had response options which were read out to participants, and one question had a free text response.

### In-depth interviews

THs were interviewed by a public health officer with prior experience of conducting interviews. The interviews were conducted in the local language, **Maithili,** following the predefined topic guide. The topic guide consisted of open-ended questions on practice, experience and finally recommendations on how to improve patient care and referral. Participants were shown a picture to identify a condition. IDIs were conducted with 29 THs, until data saturation was achieved. Interviews lasted 15 minutes on average, they were recorded and detailed notes were taken during the interview.

### Focus group discussions

Four focus group discussions (FGDs) were conducted, each with 6–10 participants. FGDs were facilitated in Maithili by the same interviewer who conducted the IDIs. The FDGs explored ways in which the TH in the groups could become more engaged in the referral care pathway for suspected MK. Using a topic guide, discussion topics were proposed following the guidelines, and the participants encouraged to share experiences, beliefs, and suggestions through interactive discussions. FGDs lasted 54 minutes on average, they were recorded and detailed notes were taken by another member of the research team.

### Data management and analysis

KAP survey data were collected on printed forms, and double-entered into a MS-Access database by two different members of the study team. All raw data were stored in a locked cabinet and databases maintained on a password-protected computer in the study office with controlled access. Both databases were compared for discrepancies, cleaned and a report generated. Data were analysed for inferential and descriptive statistics using STATA version 17. Quantitative data in this paper is presented in the form of tables and text as per themes.

Interviews and discussions were recorded on a digital voice recorder, and audio files backed up on the study computer daily. Recordings were translated and transcribed directly from Maithili to English. The notes and transcripts formed the basis of the analysis. Participants were assigned a unique study identification number and data anonymized in the transcription process. Initial interpretation included familiarization with the data. Data were coded using NVivo 12 software by SDS and verified by TM. A thematic analytical approach was used, and codes that shared certain common attributes with other codes influenced emerging themes. Impressions and interpretation of themes were discussed with native speakers and researchers. The qualitative data from this study are presented in narrative text by themes and subthemes. The presented quotations are used to illustrate dominant or varying views. 

### Ethics

The study was conducted following the tenets of the Declaration of Helsinki. The protocol was reviewed and approved by the London School of Hygiene and Tropical Medicine Observational Study Ethics Committee, UK (reference 22124) and the Nepal Health Research Council, Nepal (reference 1715). For traditional healers who were unable to read, the consent form was read out by the eye health worker. They read and further explained the points in Nepali and Maithili language depending on the language preferred by THs and gave sufficient time for them to decide on their participation. Written informed consent was obtained from the participants who were able to read, and thumb impression on the consent form was obtained from those who were illiterate, this was witnessed by a third individual (someone from the family of the participating TH). Participants gave permission for audio recordings of the interviews and focus group discussions.

## Results

### Participants

Traditional healers who participated in this study claimed to have previously provided treatment to patients with MK within the community of south-eastern Nepal. A KAP survey of MK management was conducted among 109 (male 100, female 9) healers with a mean age 65.7 years (SD 13.7). The median distance between the healer’s residence and the tertiary eye hospital (SCEH) was 24 km. The majority of THs had no formal education (67.9%) and were from Terai origin caste (68.8%) ( Table 1).

**Table 1.** Sociodemographic profile of the 109 traditional healers who participated in the KAP survey.

Characteristic	Frequency (n/109)	Percentage (%)
**Ethnic group** *		
Indigenous caste	9	8.3
Terai origin caste	75	68.8
Dalit	19	17.4
Muslim religion caste	2	1.8
Hill origin caste	4	3.7
**Gender**		
Male	100	91.7
Female	9	8.3
**Education Level**		
Illiterate	42	38.5
No formal education, but able to sign and read	32	29.4
Grade 1–4	21	19.3
Grade 5–7	4	3.7
Grade 8–10	7	6.4
Grade 10–12	2	1.8
Certificate	1	0.9

* Official Government of Nepal ethnic group terminology

In-depth interview participants were purposively selected based on responses obtained from the KAP survey to provide a range of different perspectives. We conducted 29 interviews (male 27, female 2). The mean age of the participants was 66.7 years. The majority of the interview participants were illiterate (75.9%; 22 participants) and only 2 (6.9%) had received in lower secondary school education. On average they lived 28 km from SCEH in Lahan.

FGDs were conducted to further explore challenges and referral practices of the THs. Fifty THs were invited to participate in the FGDs: 32 males agreed and 10 declined whereas none of the invited female THs agreed to participate. The participants had a mean age 65.3 years. They were divided into four groups based on the distance from the different FGD venues and TH location. Of 32 the participants, 11 (34.3%) had up to higher secondary level of education, 11 (34.3%) were able to read and sign without having any formal education and the remaining 10 (31.2%) were illiterate.

Thematic analysis of the coded transcripts and predetermined topics of the KAP survey were categorized into five themes: knowledge, treatment practices, referral practices, role of TH and improving referral systems. Knowledge, treatment practices and referral practices were specific to corneal infections.

### Theme 1 - Knowledge

Traditional healers have limited knowledge about corneal ulcers and infections. They explained that infections can be caused by a curse or witchcraft, trauma with vegetative matter, insects, or symptoms related to conjunctivitis. They believe that infection is bad for the body and is caused by impure blood and poor hygiene. One (FGD/02) said, *‘One ghost which makes people blind, it may be in his house.’*

**Figure 1.**
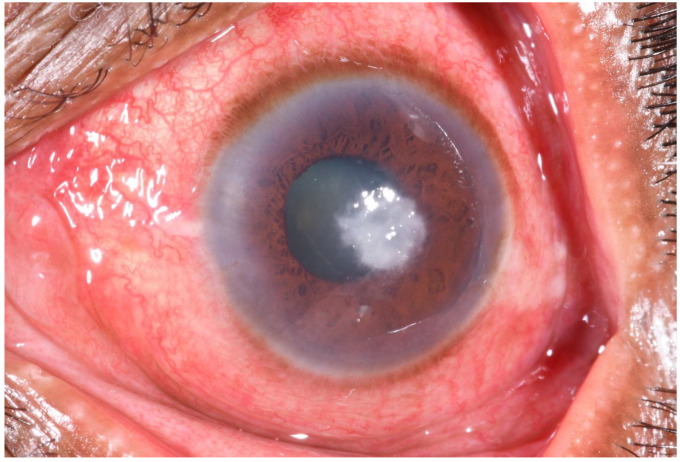
Photograph of a corneal ulcer/microbial keratitis (white mark in the eye). This image was shown to traditional healers during in the depth interviews to ask if they could identify the condition affecting the eye.

When shown a picture ( Figure 1) of a corneal ulcer during the IDI, 17/29 TH were able to recognize it as a *fulo*, *fula, or madi*—local terms for corneal ulcer. Others incorrectly identified the picture as a *motibindu*-cataract. Some of those who had prior experience with corneal ulcers stated that it initially resembles *a ‘lentil-sized white spot in the black of an eye’* (IDI/23).

**Figure 2.**
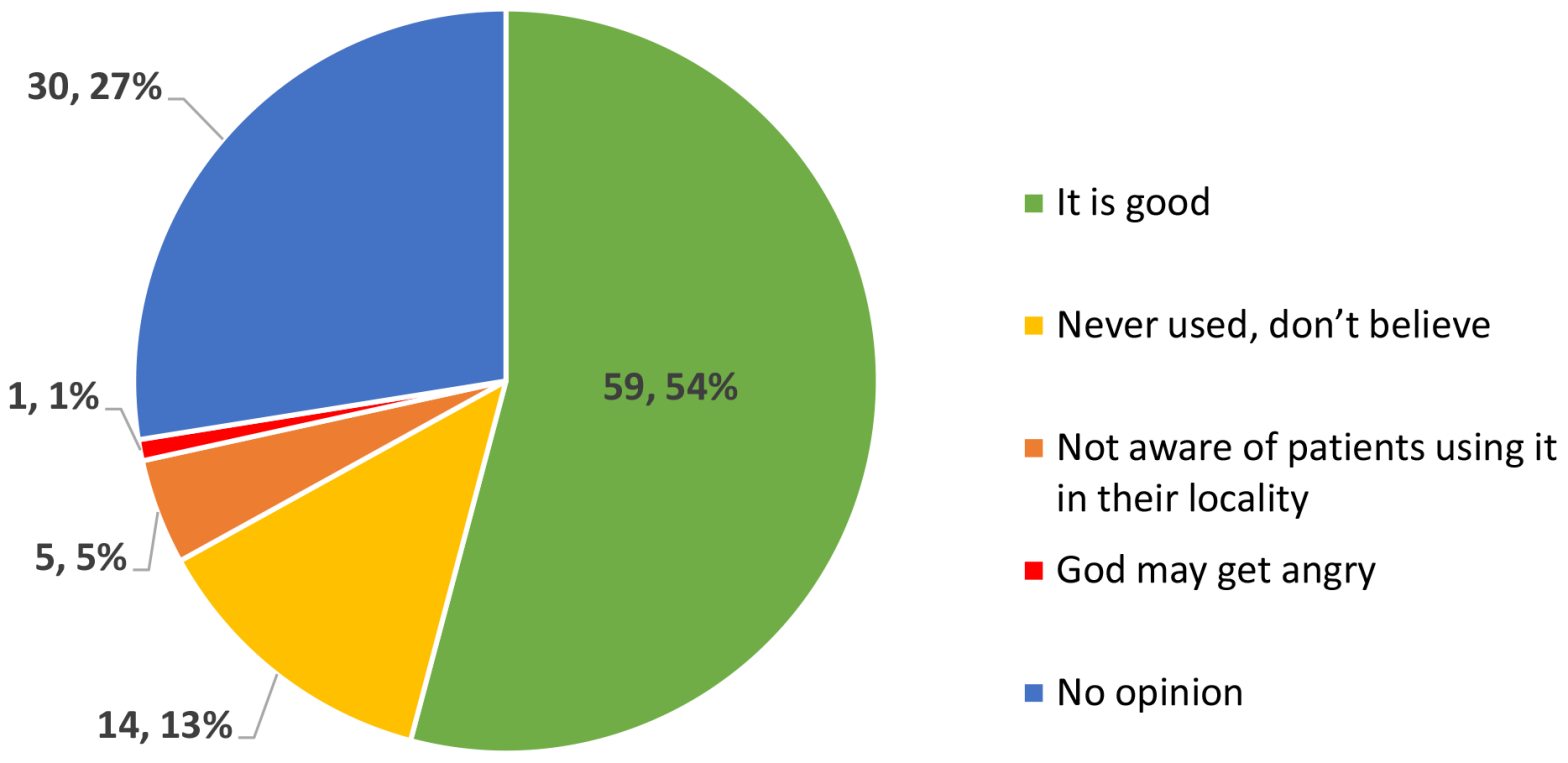
Perception of traditional healers in the KAP survey on the use of eye drops (number, %), in response to the question: “What are your thoughts on using eye drops or medicine available from pharmacy shops or medical centres to treat eye diseases?”.

Participants stated that the diseased eye gets smaller, and the patients experience pain, watering, irritation, and difficulty seeing in sunlight, and if not treated early on, it can worsen and cause blindness. They agreed among each other in FGDs that big ulcers cannot be treated at all by THs. In the KAP survey we tested their knowledge and trust in allopathic medicine and its usage in the form of eye drops. Response specific results are shown in Figure 2. Just over half of THs (54.1%) believed that using eye drops or medicines available from pharmacy shops or medical centres to treat eye diseases is good, while 27.5% of them were neutral and did not give an opinion. Approximately 13% of them had never used allopathic medicines because they do not trust them. A KAP survey participant (T0168) remarked, ‘ *God may get angry if we use modern medicines and as a result eye condition may worsen’*.

### Theme 2 - Treatment practices

The majority of THs followed spiritual or mythical practices when treating patients with MK. The most common treatment practice uses a herb that makes direct contact with the infected eye and the herb then placed into muddy soil or under water to rot. The belief is ‘ *when herb rots, the ulcer heals’*. Other common practices include chanting of mantras and prayers to God for healing. Some THs offer a *Jadi* (can be leaf, stem, root, flower, or mixture) to put into milk, cream or banana and eaten for a few weeks. Other less commonly used traditional eye medicines (TEM) included, boiled mustard oil, boiled ghee, boiled pepper along with honey or ghee, fenugreek seeds soaked with hailstone water, honey mixed with peepal tree milk, the horn of a twelve horned deer rubbed and mixed with honey, and extract of herbs mixed in a brass plate, commonly known as *Aajan.* A list of plants and other materials used by THs, their preparation and application can be found in Table 2.

**Table 2.** List of traditionally offered treatments for microbial keratitis including their preparation, application, and purpose.

Traditional eye treatments	Part of the plant	Preparation	Application	Purpose
Tulshi ( *Ocimum tenuiflorum*/holy basil)	Leaves	Plucked and given to eat	Chewed and ingested	The leaves contain spiritual mantras for healing ulcer and pain relieving
Mustard oil	Seeds	Crushed and oil is extracted	Put in the eye	To neutralise/heal MK
Kush ( *Cannabis indica*)	The plant is dried and kept in home of Hindus to perform spiritual rituals in different occasions	Dried under sun	One plant is held in hand, spiritual chanting is done, and the plant is slid multiple times from forehead to bottom of the face.	To reduce redness and pain and resolve the ulcer
Betel nut (Areca nut)	Whole nut	Nut is rubbed on a rock along with water to form a paste	The paste is applied on the temporal forehead of non-ulcered eye	To resolve ulcer and lower the pain
Pepper ( *Piper nigrum*)	Seed	Whole seeds are used to remove curses	Counted number of seeds taken in a fist, enriched with healing spiritual mantras and thrown over the face and body of the patient	To resolve ulcer and lower the pain
Pepper & Kush	Seed and plant	Dried seed and one dried kush plant	Pepper is kept in the mouth and kush is slid over the patient’s face by chanting some spiritual mantras repeatedly for some time. On finishing, the healer blows pepper air over the face of the patient.	To resolve ulcer and lower the pain
Pepper & honey/ghee	Seed and honey/ghee	Dried seed and honey/ghee	Healers keep the pepper in their mouth and chant spiritual mantras and blows air over the patient three times a week, additionally advises patient to instil a drop of honey or ghee in their eye 2–3 times daily	To resolve ulcer and lower redness
Ban Bhutka ( *Solanum nigrum*)	Leaves	Fresh green leaves crushed, and juice is filtered through soft cloth	The juice is put in the eye with ulcer	To resolve ulcer and redness
Potato ( *Solanum tuberosum*)	Whole potato	Crushed and paste is made	Potato paste is applied outside the red eye	To reduce pain and red eye
Isargaj ( *Rauvolfia serpentina*)	Seeds	Rubbed and juice is made by mixing water	The prepared juice is put in the eye	To resolve redness and pain
Kush & Betel nut and leaf	Dried kush plant, nut, and green leaf	Dried plant and nut, raw leaves of betel	Betel nut and leaves are offered to the god and kush is held in hand and spiritual mantra is chanted and slide over face	To reduce pain and red eye
Grass ( *Cynodon dactylon*)	Root	Enriched with mantras	Wrapped in a cloth and tied in the patient's neck	To resolve ulcer and subside pain
Chirchiri ( *Achyranthes aspera*)	Root	Extracted and enriched with mantra	Touches the eye with the ulcer and put under mud to rot	To resolve ulcer, reduce pain and redness
Unknown plants	Various kind of root/stem/leaf	Dried	Small piece of any plant part is broken and enriched with mantras and given to the patient to eat it along with banana or milk cream 2–3 times in a week	To reduce redness and subside pain
Pasij ( *Euphorbia royleana*)	Leaves	The leaf of the plant is broken, and milk of leaves is collected	The collected milk is put in the eye with ulcer until the eye become white	To reduce redness and subside pain
Pathalchur ( *Kalnchoe pinnata*)	Leaves	Juice is extracted	Put in the eye	To resolve ulcer and reduce redness
Fenugreek ( *Trigonella foenum*)	Seed	Seeds are roasted then crushed into powder	Chant spiritual mantra and blow the powder over the face and eye	To resolve ulcer and reduce redness
Jatamasi ( *Nardostachys jatamansi*) & Darad maida ( *Polygonatum verticillatum*)	Leaves and root	Juice is extracted from the crushed leaf and root of Darad maida is mixed together and crushed	Over the eye lid and put in the eye	To resolve ulcer, reduce pain and redness
Fenugreek & hailstone water	Seeds	Fenugreek seed is soaked over 6–8 hours, and the soaked water is mixed with hailstone water	The mixture of both is applied in the eye 2–3 times daily until the ulcer heals	To resolve ulcer and redness
Peepal ( *Ficus religiosa*) tree milk & Honey	Leaves	Milk is extracted by plucking the premature leaf and the extracted milk is then mixed with honey in equal proportion	Put in the eye	To resolve ulcer, reduce pain and redness
Water	NA	A glass of water is enriched with spiritual mantras	Patient is asked to drink it keeping the faith on God	To reduce redness and pain
Ash	NA	Use the burnt ash of incense	Rub over the forehead	To neutralise the effect of witchcraft and resolve MK with spiritual belief
Warm compress	NA	Soft cotton cloth, generally whatever patient is wearing is used	Patient is asked to blow 3–4 times into the cloth by touching the mouth, patient blows until the cloth gets warm. The warm cloth is now asked to keep over the eye with trauma and pain	To reduce pain and red eye
Ghee	NA	Melted and heated until bubbles started forming, it is then cooled and put in the eye with ulcer	Put in the eye, drop by drop 2–3 times daily	To resolve ulcer and reduce redness
Honey	Natural honey	Extracted	Put in the eye	To neutralise/heal MK
Swamp deer ( *Rucervus duvaucelii*) horn & honey	NA	The horn of swamp deer is rubbed on a rock and the generated powder is mixed with honey	Put in the eye	To resolve ulcer, reduce pain and redness

All these treatments are applied directly into the eye when cooled. One participant said, *‘I take whole black pepper in my mouth and blow their eye by chanting mantra along with God Shiva’s prayer.’* - IDI/26

Results from the KAP survey indicated 65/109 (60%) THs offer prayers for the patients to relieve pain and others (40, 37%) offer prayers combined with TEM. The majority (76, 70%) of THs said they use the root of herbs to treat eye diseases/conditions while others use other substances such as tree milk, cactus thorn, flower juice, and *Tulshi* seed extract. More than half of the participants (57, 52.7%) said that they use sun dried herbs (root, skin, leaf) and almost all (102, 95%) prepare the herb on their own. The duration of treatment is summarised in Table 3.

**Table 3.** Duration of treatment offered by 109 traditional healers to patients with eye diseases in Siraha district.

Treatment period	Frequency (n/109)	Percentage (%)
1 day	25	22.9
Up to 3 days	22	20.2
Up to 1 week	26	23.9
Up to 2 weeks	20	18.4
Up to 1 month	6	5.5
More than a month	2	1.8
Unsure	8	7.3

The reported treatment process ranged from a single day up to one month, depending upon the disease condition and the patient’s response to treatment. But a group of THs said they don’t treat patients beyond eight days, if the condition doesn’t get better, they ask patients to seek a medical consultation. They said small ulcers, the size of a mustard grain, can be treated by them but one TH (IDI/24) said *, ‘I won’t say a millet but up to half rice grain size of ulcer can be treated by us.’*

One TH said allopathic medicines are good for red eye, to which others added red eye caused by trauma needs allopathic medicine. Yet most reported that *fula* (cornea ulcer) do not respond to allopathic medicine whereas THs can treat *fula,* provided it is only small in size. They reported a few corneal ulcer cases that had healed with the treatment they gave, which had not healed with allopathic medicine. Most of them agreed that patients presenting late, with large ulcers, cannot be treated. One remarked that THs contribute toward the delay in seeking allopathic treatment for MK, in addition to poor access to transportation and the higher costs associated with care and treatment at private health facilities.

We further asked about the fees in return for the service THs offer: 78 (71%) said they don’t charge for their service whereas most of the remaining participants reported receiving some form of payment either directly asking for an amount or in kind (patient gives as they wish).

### Theme 3 - Referral practices

Referral practices reported by THs in the IDIs and FGDs varied. Many THs do not refer people at all. They expressed confidence in their practice by giving examples from their past successful treatments. Few THs said they sometimes refer patients with MK or other eye conditions to health centres (HC) or health posts (HP) and 74 (68%) reported referrals are done verbally. The majority of THs said that they don’t refer to HC or HP because they lack trust in the ability of the staff to manage eye conditions, particularly at community health posts. They feel that HPs can only manage cases of flu, fever, and cuts. Despite treatment being free, some HPs are perceived to be selective in who they provide treatment to, often using excuses of stock-outs when they do not treat. It was suggested they don’t provide services to all, but rather only to people of higher status or people whom they themselves may benefit from. Others explained that the staff responsible for outpatient visits are often called out of the HCs and HPs due to training programmes and meetings, making service provision impossible to access at times.

Some THs in FGDs reported directly referring patients to the eye hospital if an eye condition does not improve with traditional treatment within a week. Few THs preferred to treat patients with traditional remedies while the patient is simultaneously using allopathic medicines. They had an understanding that medical treatment of MK is provided by a specialist and is associated with time and cost for transportation and medical services. For this reason, some THs refer patients to HC which are located within the community.

Some of the KAP questions were designed to gauge confidence in community health services, modern medicine and referrals. Many of the THs (53, 48.6%) felt that community health posts could not treat minor eye injuries, a small number (six participants) did not answer this question ( Table 4). When asked about the treatment of emergency eye conditions, most said HPs can’t treat eye emergencies. When asked about the management of MK cases that have not healed, 64/109 (58.7%) of THs confidently said that everyone they have treated have healed so far. THs initially said everyone they treated had healed but at the same time some said they refer to hospitals if not healed. Responses to each question asked are tabulated in Table 4.

**Table 4.** Responses of 109 traditional healers on their trust in community health posts and their referral practice for patients with eye problems.

Questions and responses	Frequency (n/109)	Percentage (%)
***Do you think public health posts can treat patients with minor eye injuries?***		
Yes	43	39.5
No	53	48.6
Don’t know/Not sure	7	6.4
No answer	6	5.5
***Do you think a public health post or general hospital can treat emergency eye conditions?***		
No, they can’t	82	75.2
Only some places can	14	12.8
They can but they don’t treat	2	1.8
Same as us	2	1.8
They can	5	4.6
Don’t know	4	3.7
***What do you do if the patient has not healed after all your efforts?***		
Everyone healed	64	58.7
Refer to another traditional healer	1	0.9
Refer to a public health post	3	2.8
Refer to a pharmacy	1	0.9
Refer to a hospital (general or eye)	29	26.6
Allow patient to decide	6	5.5
No answer	5	4.6

### Theme 4 - Role of traditional healers

During the IDIs and FGDs we noted various opinions on the role of THs in treating MK. They said that it is trust which brings patients to them for further treatment and expressed confidence in what they do as well as perceived confidence of patients in THs and natural healing. *‘If it is destined to heal, it will heal’* (FGD/04).

THs expressed their dissatisfaction that some educated people do not recognise the traditional healing profession. They admitted that with the establishment of community health facilities there is a decrease in patients visiting THs. Some expressed opposition to other THs who might delay medical treatment of patients by emphasising trust in traditional treatments, resulting in worsening of the disease. One participant (FGD/04) said, *‘Some traditional healers keep them in the circle of myth.’* Participants explained that they do not want to be blamed by patients, and traditional treatment must be accountable and responsible, *‘Holding patients for long at village is sort of killing them’* (FGD/04).

### Theme 5 - Improving referral systems

THs were keen to be trained to identify eye diseases and refer patients when there is a need to do so. Majority, 47/109 (44%), of THs suggested referring cases to HP/hospital as an initiative to help reduce corneal blindness resulting from eye infections. Most of them liked the ‘train the trainer’ concept, where a small group of THs receive training and they then train other groups of THs. In one of the FGDs some suggested using training videos and photographs of the disease, while one of them asked for a book illustrating the major eye diseases. One remarked that a book could improve their relations with the hospital (TH/02).

*‘...a book which has basic details and picture of eye disease. Mention at what stage patient should be sent to eye hospital. Book will also help us to counsel patient about disease condition they have got, and they will also understand that it is not possible to treat by healers. They will then visit to eye hospital.’* (FGD/01)

On the contrary, one TH (TH/05) pointed out that a book ‘... *won’t be useful for me as I am illiterate.’*

They insisted on specialists visiting health centres at regular intervals to treat patients with eye diseases at the community level. They highlighted the need to improve their relationship with the HC and the importance of mutual respect. A meeting was proposed to discuss solutions for delays in MK treatment to which traditional healers, pharmacists, HC staff, community leaders and local government officials would be invited.

From a community perspective, despite lack of trust and confidence in what services HP can provide, many THs feel that the situation could be improved with training of HCs and THs in the management of eye conditions. The aim of which would be to increase TH confidence in referring MK patients to HPs or to an eye hospital. Although all of the THs were supportive of referring patients with MK to an eye hospital, they recognised the reality that most will not attend due to associated costs and fear. Therefore, they continue to offer traditional treatment because they consider that this is better than doing nothing.

Participants expressed a need for public awareness programs on eye disease and its severity in the community. Suggestions included broadcasts through radio or TV, loudspeaker announcements, and conducting eye camps on school premises, eye screening and eye health education. They showed respect for FCHVs and insisted on training them and motivating them to raise awareness about eye conditions as well.

## Discussion

Despite improving health systems globally, people still resort to seeking care from THs in Siraha District, south-eastern Nepal. Even though there is a tertiary level specialist eye hospital (SCEH) located an average of 24 kms away from the THs who participated in this study, many people still trust and seek care from them first. A knowledge of MK, its progression and treatment are essential, including an understanding of the pathogenesis necessitating immediate, intensive treatment under clinical supervision ^5, 16^. Most healers in this study lacked this knowledge and some reported treatment durations lasting up to a month without resolution. Patients with serious conditions like MK may delay medical treatment due to a spiritual healing practice carried out by *Dhamis*. This has been in practice for centuries in Nepal ^17^. The THs of Siraha district maintained a strong belief that eye infections may be caused by a curse or witchcraft involving an evil spirit. Typically, their ability to identify and differentiate eye conditions was poor but they seemed to be confident in their practice.

African nations like Ghana, Tanzania and South Africa have sought to preserve, promote, and integrate traditional medicines into the national health care system. In Asia it has been suggested that it is important to conserve plants used in traditional medicine for global business ^18– 21^. Japanese herbal (Kampo) formulas are ethically approved for oral and ocular use and some TEMs have proven antibiotic properties ^22, 23^. Yet, there is evidence of harm with microbial keratitis, endophthalmitis, and eye loss following from using TEM where it is unregulated ^24, 25^.

This study described a local healing practice not previously reported elsewhere. A common mythical practice of touching an infected eye with a herb (often the root of a herb) and burying the herb under mud or water to rot. It is believed that as the herb rots slowly, in a similar way the ulcer fades and the eye become clear. Many THs used a variety of other ingredients also reported by other studies ^26, 27^. The healers perceived “healed” as when the patient reported no longer having any pain, but some of them also perceived it as not returning for care assuming it might have healed. Despite sometimes providing long-term treatment (up to one month), the THs don’t ask for financial compensation. This phenomenon of extended treatment waiting until the herb rots with potentially low cost or no cost, significantly contributes toward a delay in seeking timely care in the formal health system. This likely leads to more severe MK and blindness, increasing morbidity and reducing quality of life – something observed by other studies from Nepal and Uganda ^11, 28^.

This study created an opportunity to explore traditional eye health practices and management of eye conditions at the community level. Generally, patients with MK present late to tertiary eye hospitals resulting in a poor prognosis; this is preventable with prompt presentation. Nepalese patients may encounter many barriers to using and benefiting from healthcare services, including cost of services, transportation, poverty, unemployment, education, and lack of awareness of health services available to them. People face significant obstacles when trying to access and use services, which are linked to gender, cultural norms, discrimination, and poor-quality service ^29, 30^. Similar to other LMICs, in Nepal, traditional healers and pharmacy shops are two easy points of care for patients who are experiencing barriers to accessing the formal health system. Traditional healers are well recognised and trusted within communities of Siraha District. In this study the healers acknowledged their limited understanding of different eye conditions and were interested to receive training to identify infectious eye conditions. They seemed concerned about being respected in the health system and were willing to refer cases to community health centres or eye hospital provided they are appreciated and valued as an integral part of health system. They could act as a potential gateway for MK patients in the community and the formal health system. This pathway could be realised if THs are supported to recognise and refer serious eye conditions at the time of their initial presentation, and if the primary health facilities they are asked to refer patients to are strengthened to treat common eye conditions. Improving eye care provision at the primary level will help to build THs trust in health centres.

It is important to understand the challenges faced by traditional healers and recognise their sentiments to encourage referral of suspected MK cases. This can include training them on basic identification of eye conditions e.g., painful red eye, and encourage them to refer those patients to the community health posts/centres. Establishing a mutually supportive link between THs and HCs could help bring about the change needed for THs to become an integral part of the referral system. They welcomed the concept of community discussion with stake holders, political influencers, social activists, journalist, and key community members about delayed presentation of MK and possible solutions. These gatherings are believed to make THs feel important and valued. Fostering such connections may help improve eye health services at district level and also improve the community health practice, and ultimately minimizing the problems associated with delayed healthcare seeking behaviour ^28, 31^.

This study is limited by being conducted in only one district, Siraha, therefore our findings may not be generalisable outside this area of Nepal. However, the findings highlight the importance of understanding indigenous care, existing traditional treatment practices and barriers in accessing care in the formal health system.

This study of THs supports the need for community level interventions aimed at training traditional healers and possibly pharmacy shop owners to identify corneal abrasions, painful red eyes and corneal ulcers and building supportive networks to refer to primary care facilities. An ongoing cluster randomised controlled trial in this district is looking at enhanced training of primary health centre staff to improve early detection and referral of MK [trial registry ISRCTN 95560917]. This will also assess the impact of integrating THs and pharmacies in the referral system, to counter the burden of severe microbial keratitis in this region and reducing avoidable blindness.

## Conclusion

This is the first study focused on traditional healers who are involved in treating patients with MK in Nepal. The uncoordinated referral system in the community both by THs and HCs and poor knowledge and awareness of eye health in general is a matter of concern for the Ministry of Health and policy makers, especially with respect to emergency eye conditions like MK. Traditional healers were ready to be trained to refer cases to community health centres – that are trained and equipped to provide basic eye health services - provided they are respected and recognised for their work. Better integration of THs into the health system will not only share the burden of community health volunteers but also act as a bridge for eye care into the formal health system. We believe there is a pressing need for government policy to regulate traditional medical practices in this area and strengthen primary eye care services.

## Data Availability

Interview and focus group data sets, have not been made publicly available due to the identifiable nature of the data. Mtuy, T. Nepal Traditional Healers KAP Survey dataset [Internet]. London School of Hygiene & Tropical Medicine; 2024. Available from: https://doi.org/10.17037/DATA.00003843. This project contains the following underlying data: Data file 1. (KAP dataset.) Data file 1 is available under the data sharing agreement. The KAP questionnaire (data file 3) is available within the same repository as KAP dataset, available at https://doi.org/10.17037/DATA.00003843. The topic guide with open-ended questions is available in the existing repository.
