# Supplementary material for: Role of traditional healers in the management of microbial keratitis in eastern Nepal
Source: Wellcome Open Res. 2024 Nov 4;9:295. Originally published 2024 May 30. [Version 2] doi: 10.12688/wellcomeopenres.21241.2 (PMC11541071; doi:10.12688/wellcomeopenres.21241.2)
Supplement: Peer review history

# Reviewer report: Reviewer response for version 2

**DOI:** 10.21956/wellcomeopenres.25672.r109641

**Published:** 2024-11-06

**Authors:** Bethany Mills

**Affiliations:** 1 The University of Edinburgh, Edinburgh, Scotland, UK

I have no further comments.

Is the work clearly and accurately presented and does it cite the current literature?

Yes

If applicable, is the statistical analysis and its interpretation appropriate?

Not applicable

Are all the source data underlying the results available to ensure full reproducibility?

Partly

Is the study design appropriate and is the work technically sound?

Yes

Are the conclusions drawn adequately supported by the results?

Yes

Are sufficient details of methods and analysis provided to allow replication by others?

Yes

Reviewer Expertise:

Microbial keratitis research - particularly engaging with eye-care systems in India

I confirm that I have read this submission and believe that I have an appropriate level of expertise to confirm that it is of an acceptable scientific standard.

# Reviewer report: Reviewer response for version 1

**DOI:** 10.21956/wellcomeopenres.23490.r94148

**Published:** 2024-09-12

**Authors:** Anitha Venugopal

**Affiliations:** 1 Aravind Eye Hospital, Tirunelveli, Tamil Nadu, India

Many sentences lack clarity: like Some of the participants were traced, while others could not be found or had died." This statement lacks clarity. Were efforts made to contact all participants? How many attempts were made to reach them?

 The methodology mentions that "All the questions were read aloud, and the answers given by THs were recorded in the survey record sheet." It should clarify whether the survey was self-administered or interviewer-administered. Also, there is no mention of the language used for administering the survey.

 The phrase "Purposive sampling was used to recruit for IDIs and FGDs, based on interactions with participants during the KAP survey" is vague. It does not specify what criteria were used to identify participants for the in-depth interviews and focus group discussions, nor does it provide a rationale for choosing purposive sampling.

 "Some of them were traced, while others could not be found or had died." This is a unclear description. Instead, specify how many were traced, how many could not be found, and how many had died.

 On what bases 30 community health centres were randomly selected- explain how representative the sample is?

 MSVI has not been consistently used through out the text

 no need to mention loss of an eye after monocular blindness

Is the work clearly and accurately presented and does it cite the current literature?

Yes

If applicable, is the statistical analysis and its interpretation appropriate?

Yes

Are all the source data underlying the results available to ensure full reproducibility?

No source data required

Is the study design appropriate and is the work technically sound?

Yes

Are the conclusions drawn adequately supported by the results?

Partly

Are sufficient details of methods and analysis provided to allow replication by others?

Yes

Reviewer Expertise:

Cornea, External Disease, refractive surgery

I confirm that I have read this submission and believe that I have an appropriate level of expertise to confirm that it is of an acceptable scientific standard.

# Reviewer report: Reviewer response for version 1

**DOI:** 10.21956/wellcomeopenres.23490.r94144

**Published:** 2024-09-01

**Authors:** Bethany Mills

**Affiliations:** 1 The University of Edinburgh, Edinburgh, Scotland, UK

This is a very interesting and well communicated study. The study presents a cross-sectional, mixed-methods approach to describe the self-perceived role of traditional healers in the context of microbial keratitis, in a south-eastern district of Nepal. The study aimed to capture knowledge, attitude and practices round MK management. This was conducted in three ways, including surveys, in depth interviews and focus group discussions. The study found that knowledge was limited among traditional healers, but there was a strong willingness to learn and want for better community cooperation among different levels of the healthcare services provided.  

 Source data for KAP survey are available upon request. Source data from the interviews and focus groups are not available. 

 I have a few minor comments in relation to data presentation which should be addressed: 

**Methods:**

1. It is not clear whether any of the same participants were involved in both in-depth interviews and focus group discussions. Please can you clarify within the text.

**Results:**

1. None of the KAP survey data relating to referral questioning (Q10, 11 and 15) are presented in the results section (and are not available without request). It would be beneficial to see this reported to supplement reporting from the in depth interviews and focus groups on this theme.
2. Table 2 would benefit from an additional column indicating how many respondents reported using that particular treatment in order to understand how wide-spread the particular practice is.  
3. The methods state that “presented quotations are used to illustrate dominant views” – this appears to not necessarily be the case within the results chapter. For example, figure 2 shows that only 1 participant perceived that “god may get angry” in reference to allopathic eye-drop use, however, this sentiment is presented as a quote. Please ensure the methodology and reporting align.

**I have a few final minor points of intrigue that may or may not be addressable within the discussion:**

1. I’m curious, a point is made within the text that the traditional healers contradicted themselves between stating that everyone they treat was healed, and at the same time, they referred non-healers. Was there any indication about what “healed” meant to the traditional healers in this context?
2. As per the methodology, participants were recruited from an equal proportion of rural, urban and semi-urban regions. The sample size may be too small to draw any definitive conclusions, but were any general differences in attitudes, practices and knowledge observed between these different settings?  
3. Do you consider that there may be any potential biases or limitations introduced based on the gender balance within the study? Is it proportional for the region that >90% of traditional healers in the region are male?

**Other:**

 While the KAP survey questionnaire is available in the supplementary information, the topic guide with the open-ended questions is not. Is it possible to make this available?

Is the work clearly and accurately presented and does it cite the current literature?

Yes

If applicable, is the statistical analysis and its interpretation appropriate?

Not applicable

Are all the source data underlying the results available to ensure full reproducibility?

Partly

Is the study design appropriate and is the work technically sound?

Yes

Are the conclusions drawn adequately supported by the results?

Yes

Are sufficient details of methods and analysis provided to allow replication by others?

Yes

Reviewer Expertise:

Microbial keratitis research - particularly engaging with eye-care systems in India

I confirm that I have read this submission and believe that I have an appropriate level of expertise to confirm that it is of an acceptable scientific standard, however I have significant reservations, as outlined above.
